# Exploring the statistical fragility of creativity studies using the alternate uses task: A systematic review protocol

**DOI:** 10.1371/journal.pone.0333910

**Published:** 2025-10-07

**Authors:** Vikram Arora, Alex Thabane, Adam Sutoski, Kim Madden, Mohit Bhandari

**Affiliations:** 1 Department of Health Research Methods, Evidence, and Impact, McMaster University, Hamilton, Ontario, Canada; 2 Temerty Faculty of Medicine, University of Toronto, Toronto, Ontario, Canada; 3 Faculty of Medicine and Health Sciences, McGill University, Montreal, Québec, Canada; 4 Department of Surgery, McMaster University Medical Center, Hamilton, Ontario, Canada; Kalinga Institute of Medical Sciences, INDIA

## Abstract

**Introduction:**

The Alternate Uses Task (AUT), where participants generate multiple novel uses for everyday objects, is one of the most widely used measures of creative potential. While many creativity studies report statistically significant effects on AUT outcomes, the robustness of these findings has not been systematically evaluated. Given concerns about over-reliance on *p*-values and the risk of fragile results, this systematic review aims to assess the statistical fragility of AUT-based findings using the continuous fragility index (CFI).

**Methods:**

This protocol has been registered in Open Science Framework (DOI: 10.17605/OSF.IO/KFQGU). Eligible studies will include two-arm experimental designs (randomized or non-randomized) that report statistically significant between-group differences on AUT outcomes. We plan to conduct a comprehensive search of databases, including Medline, Embase, PsycInfo, and ERIC, up to July 21, 2025. Two reviewers will independently screen studies and perform data extraction. The primary outcome will be the median CFI across included studies, calculated using a simulation-based method developed for continuous outcomes. As a secondary objective, we plan to conduct a multivariable linear regression and subgroup analyses to explore study-level predictors of fragility.

**Dissemination:**

We will disseminate results through publication in a peer-reviewed journal and presentations at academic conferences. The findings aim to raise awareness of statistical fragility in creativity research and encourage stronger reporting and methodological practices for studies using the AUT.

## Introduction

Divergent thinking tests, which evaluate the ability to generate multiple novel ideas to open-ended problems, are foundational in creativity research [1,2]. One of the most widely used divergent thinking tests is the Alternate Uses Task (AUT), introduced in 1967 by the father of modern creativity research, J. P. Guilford [3]. The AUT asks participants to generate as many alternative uses for a common object (e.g., a brick or a paperclip) as possible within a time limit, scoring their responses for fluency (i.e., number of ideas), originality (i.e., uncommonness of ideas), and flexibility (i.e., diversity of idea categories). To date, the AUT has been used in hundreds of studies as a measure of creative potential, with many of these studies involving statistical hypothesis testing.

Despite staunch criticism over the last century, the practice of null hypothesis significance testing, with the customary, dichotomous 0.05 cut-off, continues to be widespread. Its limitations are well-known [4–6], one of which is the issue of ‘fragility,’ where a small change in the data can erase its statistical significance. This is particularly concerning, as a finding that narrowly meets the threshold to reject the null hypothesis (e.g., *p* = 0.045) is interpreted completely differently from one that just misses it (e.g., *p* = 0.055), even if the underlying effect sizes are similar and a slight change in the dataset could lead to a failure to reject the null hypothesis [7].

In response to these concerns, researchers have proposed additional metrics to assess the robustness of statistical test results. One such metric is the fragility index (FI), originally created by Feinstein (1990) and later popularized by Walsh et al. (2014) [8,9]. The FI quantifies the robustness of a statistically significant dichotomous outcome by determining the minimum number of participants with an event in one group whose status would have to switch (from event to non-event) to make a statistically significant between-group difference no longer statistically significant. For instance, if a statistically significant outcome has an FI of 1, it means that changing the outcome of just one participant would nullify the statistical significance. Such a result would be considered extremely fragile, as its statistical significance depends on a single data point. In contrast, a larger FI indicates that the result would remain significant even when several events were changed, representing a more robust finding. Furthermore, comparing the FI to the number of participants lost to follow-up in a longitudinal study can also provide important evidence on the risk of bias due to attrition in a study. Ultimately, the FI provides an intuitive gauge of evidence stability that complements the binary significance test.

A key limitation of the traditional FI is that it only applies to dichotomous outcomes. This has led to the exclusion of a large portion of studies in fragility analyses; prior methodological reviews reported having to omit between 25% and 82% of studies because they did not report any binary outcomes [10–12]. This is a particular concern in creativity research, as virtually all measures of creativity, including the AUT, are continuous variables, rendering the traditional FI unusable. To address this gap, Caldwell and colleagues introduced the continuous fragility index (CFI), extending the fragility index methodology to continuous outcomes [11]. The CFI is determined by an algorithm that iteratively shifts individual data points between groups until a significant between-group difference becomes non-significant. Similar to the traditional FI, it determines how many individual observations would need to be “moved” to undermine a statistically significant difference in a continuous outcome. This method enables researchers to quantify the robustness of findings for continuous measures, which is particularly relevant for fields such as creativity research, where continuous data are the norm.

While studies of fragility are common in the medical domain [13,14], the assessment of the fragility of study findings in creativity research is completely lacking. We therefore propose a study that explores the fragility of creativity research studies using the AUT, one of the most widely used tests of creative potential. To our knowledge, our study will be the first to examine the fragility of findings in the creativity literature. The results of this study will provide evidence of the robustness, or lack thereof, of statistically significant findings in the existing AUT literature, and by extension, the creativity literature as a whole.

## Methods

This protocol has been registered on the Open Science Framework (DOI: 10.17605/OSF.IO/KFQGU). It has also been prepared and reported in accordance with the Preferred Reporting Items for Systematic Review and Meta-Analysis Protocols (PRISMA-P) guidelines (S1 File) [15].

### Review team

The review team consists of a creativity research expert with PhD training in health research methods, a PhD in clinical epidemiology with experience designing and conducting experimental studies, a clinician-scientist with leading experience in health research in the field of orthopaedics, an MD candidate with training in research methods and prior experience conducting methodological studies of creativity, and an MSc candidate with training in research methods and prior experience with systematic reviews and methodological studies.

### Study objectives

The primary objective is to determine the fragility of statistically significant test results derived from AUT testing. The secondary objective is to identify factors associated with fragility and explore the difference in fragility between randomized and non-randomized trials.

### Selection criteria

Studies must meet the following eligibility criteria to be included: (1) two-group studies that report a statistically significant result (*p* < 0.05) on the AUT score; (2) published in a peer-reviewed journal; and (3) reporting details on the sample size, mean values, and spread statistics (e.g., standard deviation, confidence interval, range) for the significant outcome for both arms. The exclusion criteria will be: (1) letters, reviews, meta-analyses, protocols, and abstract-only publications; and (2) studies utilizing crossover designs and within-subjects designs. Any studies not published in English will be translated with Google Translate.

### Search strategies

We will systematically search Medline, Embase, PsycInfo, and ERIC from inception to July 21, 2025. In each database, we will perform comprehensive searches for all studies that included different permutations of the keyword ‘AUT,’ such as ‘alternate uses,’ ‘alternative uses,’ and ‘Guilford’s uses,’ in order to capture all studies that referenced the Alternate Uses Task. The full search strategy for each database is included in S2 File. Additionally, we will conduct a manual search of the reference lists of all included studies and relevant review articles to identify any additional eligible studies that may not have been captured in the initial database search.

### Screening

Two reviewers will independently screen the titles and abstracts of all records identified in the search. Any disagreements will be resolved through discussion, and if needed, a third reviewer will be consulted. Full texts will then be retrieved for studies deemed potentially eligible. The same dual-reviewer process will be followed during full-text screening. We will use Covidence (Melbourne, Victoria, Australia) as the platform for screening studies [16]. The results of our study screening procedure will be presented using a PRISMA flow chart.

### Data extraction

We will develop a standardized data extraction template to extract the relevant data from eligible studies. Two reviewers will independently extract the data, with any discrepancies resolved by discussion to achieve consensus or adjudication by a third reviewer. Extracted data will include study characteristics (journal name, journal impact factor, country of conduct, year of publication, number of citations), type of intervention, sample size, sample demographics, *p*-value, and the mean and standard deviation for the AUT scores. If the standard deviation is not available, we will extract other available measures of spread, such as standard error or range.

### Continuous fragility index

The continuous fragility index (CFI) for continuous outcomes estimates the number of data point shifts needed to render a result non-significant (*p* ≥ 0.05). To determine CFI, Caldwell and colleagues described an iterative algorithm that performs a Welch’s t-test on two datasets representing a continuous outcome variable [11]. The process begins by identifying the group with the higher mean, and the data point closest to the mean in the higher-scoring group is transferred to the lower-scoring group. Then, the Welch’s t-test is recalculated. This process of shifting data points and recalculating is repeated, one data point at a time, until the *p*-value is above 0.05. The CFI is defined as the number of data points that must change to cross this statistical threshold. However, this method requires complete individual participant data. Most published studies only report summary statistics, such as group means, standard deviations, and sample sizes. To address this limitation, Caldwell et al. developed a simulation-based approximation method that reconstructs candidate datasets assuming a normal distribution around the reported group statistics, allowing researchers to estimate the CFI using only aggregate data [11].

For this review, CFI values will be calculated using an online calculator based on the simulation methodology established by Calwell and colleagues. For each study, the required inputs will include the sample size, mean, and standard deviation for each of the two groups. If studies report spread statistics other than standard deviation, such as standard error or the range, we will estimate the standard deviation using appropriate conversion methods [17,18]. To minimize bias introduced by random data generation, each CFI calculation will be repeated across five simulation iterations. We will report and use the average CFI for all further analyses. A tolerance level of 0.01 will be set to ensure that simulated datasets closely match the original summary statistics reported in each study.

### Risk of bias assessment

Two reviewers will independently assess the risk of bias for each included study using standardized tools appropriate for the study design. Discrepancies between reviewers will be resolved through discussion; if a consensus cannot be reached, we will consult an additional reviewer.

For randomized studies, we will use the Cochrane Risk of Bias 2 tool [19], which evaluates five domains of potential bias: (1) bias arising from the randomization process, (2) bias due to deviations from intended interventions, (3) bias due to missing outcome data, (4) bias in measurement of the outcome, and (5) bias in selection of the reported result. For each domain, studies will be rated as having a low risk of bias, some concerns, or a high risk of bias, with an overall risk of bias judgment derived according to Cochrane guidelines.

For non-randomized studies, we will use the Risk Of Bias In Non-randomized Studies – of Interventions (ROBINS-I) tool [20]. This tool evaluates seven domains: (1) bias due to confounding, (2) bias in selection of participants, (3) bias in classification of interventions, (4) bias due to deviations from intended interventions, (5) bias due to missing data, (6) bias in measurement of outcomes, and (7) bias in selection of the reported result. Each domain will be rated as low, moderate, serious, or critical risk of bias, or no information if details are insufficient for judgment. Each study will be classified as having a low, moderate, serious, or critical risk of bias, or as having no information if the details are insufficient for judgment.

We will present groupings of overall risk of bias in tables and prepare visualizations (e.g., traffic light plots) to facilitate a clear presentation of the overall risk of bias across studies. For the purposes of further analyses, we will consider a serious or critical risk of bias in non-randomized studies to be equivalent to a high risk of bias in randomized studies. Additionally, the moderate and some concerns categories for non-randomized and randomized trials, respectively, will be considered equivalent, allowing us to combine data from both study designs.

### Statistical analysis

To meet our primary objective, we will report the CFI of included studies descriptively, presenting the median and interquartile range (IQR). We will also graphically show the distribution of CFIs using a histogram. Additionally, we will report the median CFIs with IQRs for the following sub-groups in a table: journal impact factor, year of publication, country of conduct, number of citations, type of intervention, and risk of bias classification.

For the secondary objective, we will perform multivariable linear regression to explore factors associated with statistical fragility in AUT studies. Specifically, we will consider journal impact factor, year of publication, sample size, country of conduct, the number of citations, and risk of bias classification. Based on recommended guidelines [21], we will only include the categorical variables (i.e., country of conduct and risk of bias classification) if the events per variable threshold of 10 is met. For the model, we will report beta regression coefficients along with the associated 95% confidence intervals. If needed, we will transform the CFI values to meet the assumptions of linear regression modelling [22]. For our final secondary objective, we will perform a Mann-Whitney U test to determine if there is a statistically significant difference in the CFI of randomized and non-randomized trials.

### Study timeline

We plan to begin this systematic review in September 2025, when we will conduct all database searches. Title and abstract screening, followed by full-text screening and data extraction, will be completed by the end of October 2025. We plan to prepare the first draft of the manuscript by February 2026, with final submission to a peer-reviewed journal targeted for April 2026.

## Ethics and dissemination

As this study involves only secondary analysis of aggregate data from published studies, we do not require ethics approval.

Results will be submitted to a peer-reviewed journal for publication and the authors will share the findings at local, national, and international academic conferences. We aim to raise awareness of fragility as a relevant dimension of methodological quality in creativity research and to inform best practices for the design and reporting of future studies that use the AUT.

## Conclusion

This systematic review will be the first to apply the CFI to studies in the creativity research domain, specifically focusing on experiments using the AUT. By quantifying the statistical fragility of significant AUT findings, this study will provide valuable insight into the robustness and reliability of reported effects in creativity interventions.

## Supporting information

S1 File PRISMA-P 2015 Checklist. (PDF)

S2 File Search Strategy for Databases. (PDF)
