# Supplementary material for: Exploring the statistical fragility of creativity studies using the alternate uses task: A systematic review protocol
Source: PLoS One. 2025 Oct 7;20(10):e0333910. doi: 10.1371/journal.pone.0333910 (PMC12503252; doi:10.1371/journal.pone.0333910)
Supplement: S2 File — (PDF) [file pone.0333910.s002.pdf]

S2 File: Search Strategy for Databases

Embase
("Alternate Uses" or "Alternative Uses" or "Unusual Uses" or "Object Uses" or "Guilford's Uses" or "AUT").mp.

APA PsycInfo
("Alternate Uses" or "Alternative Uses" or "Unusual Uses" or "Object Uses" or "Guilford's Uses" or "AUT").mp.

Ovid MEDLINE(R) ALL
("Alternate Uses" or "Alternative Uses" or "Unusual Uses" or "Object Uses" or "Guilford's Uses" or "AUT").mp.

ERIC
("Alternate Uses" OR "Alternative Uses" OR "Unusual Uses" OR "Object Uses" OR "Guilford's Uses" OR "AUT") AND PEER(yes)
